# Reactivity of Hydrogen-Related Electron Centers in Powders, Layers, and Electrodes Consisting of Anatase TiO_2_ Nanocrystal Aggregates

**DOI:** 10.1021/acs.jpcc.1c01580

**Published:** 2021-06-22

**Authors:** Juan Miguel Jiménez, Daniel Perdolt, Thomas Berger

**Affiliations:** † Department of Chemistry and Physics of Materials, University of Salzburg, Jakob-Haringer-Strasse 2a, A-5020 Salzburg, Austria

## Abstract

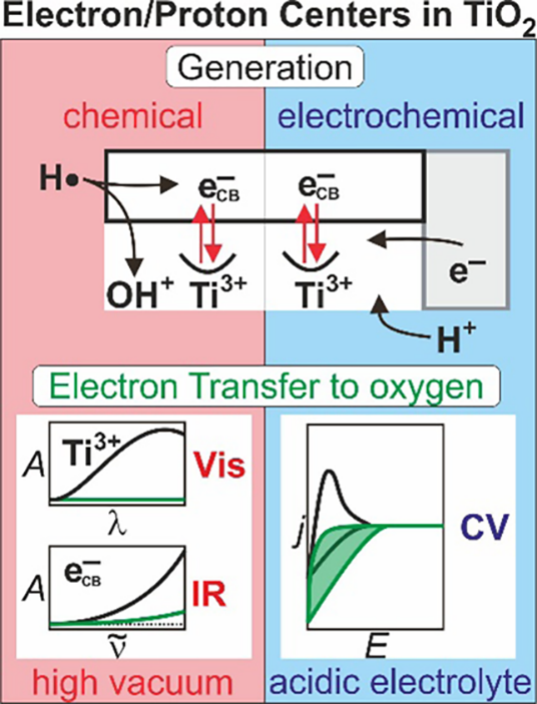

Anatase TiO_2_ nanoparticle aggregates were used as model systems for studying at different water activities the reactivity of electron centers at semiconductor surfaces. The investigated surface conditions evolve from a solid/vacuum interface to a solid/bulk electrolyte interface. Hydrogen-related electron centers were generated either chemically—upon sample exposure to atomic hydrogen at the semiconductor/gas interface—or electrochemically—upon bias-induced charge accumulation at the semiconductor/electrolyte interface. Based on their corresponding spectroscopic and electrochemical fingerprints, we investigated the reactivity of hydrogen-related electron centers as a function of the interfacial condition and at different levels of complexity, that is, (i) for dehydrated and (partially) dehydroxylated oxide surfaces, (ii) for oxide surfaces covered by a thin film of interfacial water, and (iii) for oxide surfaces in contact with a 0.1 M HClO_4_ aqueous solution. Visible (Vis) and infrared (IR) spectroscopy evidence a chemical equilibrium between hydrogen atoms in the gas phase and—following their dissociation—electron/proton centers in the oxide. The excess electrons are either localized forming (Vis-active) Ti^3+^ centers or delocalized as (IR-active) free conduction band electrons. The addition of molecular oxygen to chemically reduced anatase TiO_2_ nanoparticle aggregates leads to a quantitative quenching of Ti^3+^ centers, while a fraction of ∼10% of hydrogen-derived conduction band electrons remains in the oxide pointing to a persistent hydrogen doping of the semiconductor. Neither trapped electrons (i.e., Ti^3+^ centers) nor conduction band electrons react with water or its adsorption products at the oxide surface. However, the presence of an interfacial water layer does not impede the electron transfer to molecular oxygen. At the semiconductor/electrolyte interface, inactivity of trapped electrons with regard to water reduction and electron transfer to oxygen were evidenced by cyclic voltammetry.

## 1 Introduction

Charge transfer across solid/solid, solid/liquid, or solid/gas interfaces in nanostructured semiconductor materials is exploited in several technologies. The thermodynamic and kinetic details of the charge transfer depend critically on the chemical and structural properties of the interfaces involved. Under application-relevant conditions (e.g., in the presence of a surrounding electrolyte), interfacial conditions of these high surface area materials strongly differ from the situation at solid/vacuum or solid/gas interfaces, which are frequently investigated in model studies. At the same time, the elucidation of the impact of interfacial composition on the energetics and dynamics of interfacial charge transfer is of prime importance for the optimization of materials’ functional properties. However, related knowledge gain is challenging and requires the use of well-defined and tunable model systems, the study of relevant model reactions, and the availability of suitable analytical tools.

The reactivity of electron centers in semiconductor materials is exploited in many technological applications, including photocatalysis and electrocatalysis. Reactive electrons can be generated in a semiconductor nanostructure via different processes. Electron–hole pairs, for instance, are formed in the semiconductor bulk upon supra band gap excitation. This physical process constitutes one prerequisite for a subsequent photocatalytic event at the semiconductor/gas or semiconductor/electrolyte interface.^1^ Alternatively, charge can be injected into the semiconductor via chemical,^2^ photochemical,^3^ or electrochemical processes^4^ as exploited in sensitized photoelectrochemical cells, batteries, or electrochromic devices.

Extensive theoretical and experimental efforts have been made to characterize impurity donors such as hydrogen in metal oxides.^5−7^ In this context, infrared (IR) spectroscopy constitutes a very useful method, as it allows for the detection not only of hydrogen vibrational modes, but also of free and shallow trapped electrons.^7−10^ A broad signal in the IR range, monotonically increasing toward lower wavenumbers, was observed under high-vacuum conditions after exposure of TiO_2_ nanoparticles to atomic hydrogen or upon hydrogen dissociation and spillover on Au/TiO_2_ nanoparticles, respectively.^11,12^ In both cases, hydrogen atoms were expected to diffuse into the TiO_2_ bulk and to donate an electron to shallow trapped states just below the conduction band. Electron excitation from these states to the conduction band and inter-conduction-band transitions (Drude-type absorption) were proposed to contribute to the broad IR signal.^11,12^ Interestingly, such IR signals were detected not only under high-vacuum conditions but also upon band gap excitation of TiO_2_ in contact with aqueous solutions of hole acceptors^13,14^ and upon cathodic polarization in acidic aqueous electrolytes.^14−17^

In addition to the IR signal, a broad absorption in the visible range is observed upon charge accumulation in TiO_2_ nanoparticle ensembles and has been interpreted in terms of d-d transitions of Ti^3+^ centers, that is, electrons localized in band gap states.^17−24^ Electron paramagnetic resonance spectroscopy has evidenced the presence of Ti^3+^ species in TiO_2_ nanoparticles after negative polarization in acidic aqueous solution^19^ and after reductive treatment with atomic hydrogen.^25^ In line with these interpretations, calculations have confirmed that the exposure of TiO_2_ to atomic hydrogen produces Ti^3+^ species as a result of H atom dissociation into a proton, bound to a lattice oxygen, and an extra electron.^5^

Electrochemical accumulation of electrons in semiconductor electrodes in contact with aqueous electrolytes is related to H atom ionization in vacuum insofar as electron localization and proton adsorption/intercalation occur in parallel in both cases.^26^ In acidic electrolytes, electrochemical electron accumulation is compensated mainly by proton adsorption at the oxide surface^27^
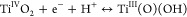
1

Because of the small size of protons, charge compensation may take place also via ion insertion into subsurface regions of the nanocrystals. In such a case, proton diffusion in the oxide bulk is the rate-determining process in both charging and discharging, possibly leading to a transient doping of the semiconductor (electrochemical hydrogen doping).^15,16,28^

In the present study, we exploit the donor properties of atomic hydrogen to chemically charge under high-vacuum conditions powders and immobilized layers of TiO_2_ nanoparticle aggregates and evaluate the chemical reactivity of accumulated electrons toward acceptor species.^2,25^ In particular, atomic hydrogen is used to generate charged states on clean surfaces while preserving the metal-to-oxygen ratio of the semiconductor. This allows for studying in a systematic way the impact of the interface condition on electron transfer reactions via a stepwise increase of interface complexity starting from a well-defined reference. Aiming at a stepwise build-up of a charged semiconductor/electrolyte interface, we adsorbed water onto the surface of chemically reduced TiO_2_ aggregates and studied electron transfer reactions in the presence of a thin interfacial water film. Finally, we complemented the study of electron transfer processes at the solid/gas interface by evaluating the reactivity of electrochemically accumulated electron/proton centers. Using hydrogen-related electronic states, we thus probe the electronic properties of defects in semiconductor nanostructures and their reactivity in different environments and at different levels of complexity (including well-defined model conditions, that is, high-vacuum and application-relevant conditions, i.e., aqueous electrolytes). In addition, careful sample synthesis and processing allows us to make the same nanoparticle-based material accessible to analysis by different analytical methods thereby gaining a comprehensive view of the properties of hydrogen-related electron centers in the same material but at very different water activities. The strategy followed in this paper aims at making a step toward bridging the gap between model studies and application. This is a challenging task for all sample types and especially for high surface area, nanoparticle-based materials.

## 2 Experimental Section

### 2.1 Chemicals

Titanium(IV)isopropoxide (99.999%) and perchloric acid (70% w/w in water) were purchased from Sigma Aldrich and used without further purification. Ultrapure water (18 MΩ cm) was obtained using a Milli-Q water purification system (Merck Millipore) and was cleaned from dissolved gases either using the freeze–pump–thaw method (visible (Vis) and IR-spectroscopy investigation of water adsorption onto TiO_2_ powders and layers) or by bubbling N_2_ through the aqueous electrolyte (cyclic voltammetry).

### 2.2 Chemical Vapor Synthesis of Anatase TiO_2_ Nanoparticle Powders

Anatase TiO_2_ nanocrystals were prepared by metal–organic chemical vapor synthesis (MOCVS) based on the decomposition of titanium(IV) isopropoxide at *T* = 1073 K in a hot wall reactor system.^29,30^ For purification, the obtained powder samples were subjected to thermal treatment under high-vacuum conditions (*p* < 10^–5^ mbar). First, the powder sample was heated to *T* = 600 °C using a rate of *r* ≤ 5 °C min^–1^. Subsequent oxidation with O_2_ at this temperature was applied to remove organic remnants from the precursor material and to guarantee the stoichiometric composition of the oxide.

### 2.3 Ensembles of Anatase TiO_2_ Nanoparticle Aggregates

The resulting particle powder was used as the precursor for slurry preparation. The TiO_2_ nanoparticle powder (0.2 g) was ground in ultrapure water (Millipore, 18.2 MΩ cm, 1.28 mL) in the absence of any additives to avoid the adsorption of organic molecules on the high surface area material. The carbon content of water-treated and subsequently dried anatase TiO_2_ nanocrystal samples, which result from the adsorption of ubiquitous carbon-containing species during sample handling in ambient air and in water, was estimated in a previous study to correspond to ∼0.3% of a monolayer at the surface of the nanoparticles.^31^

Previous studies^31,32^ have shown that both the size and the crystal structure of vapor-phase grown and thermally processed anatase TiO_2_ nanoparticles are preserved, if the particle powder is dispersed in pure water, dried at room temperature, and finally annealed at *T* ≤ 450 °C in air. Aggregate formation was therefore performed for all sample types (i.e., powders, layers, and films/electrodes) at *T* = 450 °C in air (1 h). Thermal processing at this temperature results in the formation of particle/particle interfaces imparting electronic conductivity to the particle network.^32^

#### 2.3.1 Aggregate Powder

For the preparation of a loose aggregate powder, the aqueous nanoparticle slurry was placed in a porcelain dish, dried at room temperature, and thermally annealed at *T* = 450 °C in air. The resulting sample was carefully ground in a mortar to homogenize the aggregate powder.

#### 2.3.2 Aggregate Layers

Supported nanoparticle aggregates were prepared by spreading the aqueous nanoparticle slurry onto a tungsten mesh (Alfa Aesar, tungsten gauze, 100 mesh woven from 0.0509 mm diameter wire). The sample was then dried at room temperature and thermally annealed at *T* = 450 °C in air. After sintering, the film thickness accounts for 300 ± 50 μm.

#### 2.3.3 Aggregate Films/Electrodes

The aqueous nanoparticle slurry was spread by doctor blading onto fluorine doped tin oxide (FTO) coated glass (Pilkington TEC 8, resistance 8 Ω/□). The resulting films were dried at room temperature and thermally annealed in air at *T* = 450 °C. After sintering, the mean film thickness accounts for 10 ± 3 μm. A copper wire was attached to the conducting substrates with silver epoxy. The contact area and the uncovered parts of the substrate were finally sealed by epoxy resin.

### 2.4 Diffuse Reflectance Vis-Spectroscopic Study of Loose Aggregate Powders and Aggregate Films

The aggregate powder was filled into an alumina ceramic boat and placed in a quartz glass tube. Alternatively, aggregate films deposited on FTO-coated glass (i.e., electrodes) were directly inserted into the quartz glass tube. The tube was connected to a dedicated high-vacuum system, which allows in addition to Vis-spectroscopic experiments in diffuse reflectance mode for (i) thermal sample activation under high-vacuum conditions (*p* ≤ 10^–5^ mbar) and in oxygen atmosphere, (ii) chemical reduction of the samples by atomic hydrogen, and (iii) sample exposure to defined gas and vapor atmospheres (Figure S3a).

The process of aggregate formation as described in Section 2.3 and sample transfer into the high-vacuum reactor are carried out under atmospheric conditions. In order to remove adsorbates from the aggregates’ surface, samples were once more subjected to a thermal activation procedure under high-vacuum conditions as well as in a defined oxygen atmosphere. In particular, samples were heated under high-vacuum conditions to *T* = 450 °C (temperature ramp: 10 °C·min^–1^) and thermally annealed at this temperature under high-vacuum conditions (30 min) and in an oxygen atmosphere (*p*[O_2_] = 100 mbar, 30 min). Afterward, the sample was cooled down to room temperature and then high-vacuum conditions were reestablished. We will refer to a sample, which was subjected to such an activation procedure as an *activated sample*. Activated samples were used as the reference for all Vis-spectroscopic experiments, that is, all Vis spectra are referenced to the spectrum of an activated aggregate powder or film, respectively. Vis spectra were recorded in diffuse reflectance mode using a fiber optic system consisting of an HR4000 spectrometer (Ocean Optics) and a 150 W Xe lamp (Oriel Instruments). Five scans (integration time: 6 s) were accumulated in order to obtain spectra with a reasonable signal-to-noise ratio resulting in a sampling rate of 2 spectra per minute.

### 2.5 IR Spectroscopy Study of Immobilized Aggregate Layers

For transmission Fourier-transform infrared spectroscopy, a high-vacuum cell developed by J. T. Yates Jr. and co-workers^33^ was used and for this purpose aligned in the optical path of the IR beam of a Bruker Tensor 27 spectrometer system and connected to an atomic hydrogen generator unit (Figure S3b). The resolution was 4 cm^–1^, and 100 interferogram scans were averaged to guarantee a reasonable signal-to-noise ratio.

IR spectra were recorded at room temperature. Bands between 3000 and 2800 cm^–1^, which result from organic contamination of the spectrometer’s optical components, were observed and represent sample-independent artifacts. Therefore, corresponding data points in this spectral region have been removed and replaced by a dashed line.

The tungsten mesh carrying the aggregate layer was inserted into the IR spectroscopy reactor. After the establishment of high-vacuum conditions, the supported aggregate layer was activated (following the protocol described in Section 2.4), that is, samples were heated (by resistive heating of the supporting tungsten mesh) under high-vacuum conditions to *T* = 450 °C (temperature ramp: 10 °C·min^–1^) and thermally annealed at this temperature under high-vacuum conditions (30 min) and in an oxygen atmosphere (*p*[O_2_] = 100 mbar, 30 min). Afterward, the sample was cooled down to room temperature and then high-vacuum conditions were reestablished. Again, we will refer to a sample, which was subjected to such an activation procedure as an *activated sample*.

### 2.6 Chemical Reduction of Loose Aggregate Powders and Aggregate Layers by Atomic Hydrogen

Atomic hydrogen was generated in the Vis- and IR-spectroscopy reactors via homolytic splitting of molecular hydrogen at the surface of a coiled tungsten filament at *T* ∼ 2000 K (hydrogen background pressure *p*[H_2_] = 10^–3^ mbar).^33^ The sample was optically shielded from the heated tungsten filament to avoid sample heating when operating the tungsten coil. The temperature of aggregate layers was monitored in situ (via a thermocouple connected to the tungsten mesh carrying the sample) and no significant temperature increase was observed upon sample exposure to atomic hydrogen. For all samples reported here, we did not observe any spectral changes (in the Vis and IR) upon sample exposure to molecular hydrogen (i.e., when the tungsten filament was not heated).

### 2.7 Cyclic Voltammetry Study of Immobilized Aggregate Films/Electrodes

Measurements were performed in a standard three electrode electrochemical cell. Electrolytes were purged from O_2_ by bubbling N_2_ through the electrolyte (i.e., 0.1 M HClO_4_ aqueous solution). Alternatively, O_2_ was bubbled through the electrolyte to maximize the concentration of dissolved oxygen. All potentials were measured against and are referred to a Ag/AgCl/KCl (3 M) electrode (BasInc). A Pt wire was used as a counter electrode. Measurements were performed with a computer-controlled Autolab PGSTAT302N potentiostat. The current densities are given on the basis of the geometric area.

## 3 Results and Discussion

### 3.1 Chemical Charge Accumulation and Electron Transfer at the Solid/Gas Interface

Excess electrons are generated at the surface of anatase TiO_2_ aggregates upon the dissociation of atomic hydrogen into protons and electrons. The excess electrons can be either localized, forming Ti^3+^ centers
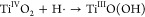
2or delocalized in the conduction band
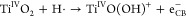
3

In the following, Vis spectroscopy is used to investigate the generation and reactivity of Ti^3+^ centers, and conduction band electrons are tracked by IR spectroscopy (Sections 3.1.3−3.1.4).

#### 3.1.1 Chemical Reduction of TiO_2_ Aggregate Powder by Atomic Hydrogen: Vis-Active Ti^3+^ Centers

**Figure 1:**
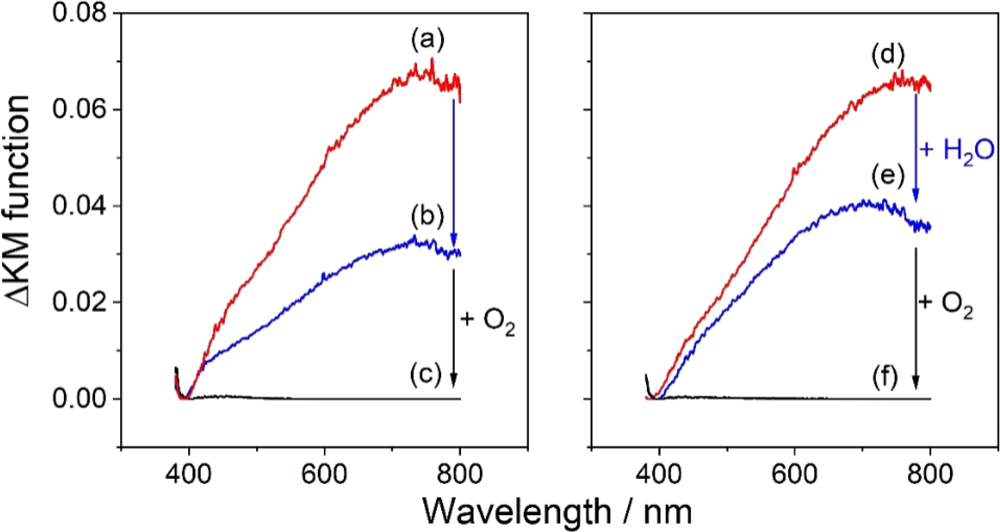
(a–c) Diffuse reflectance Vis spectra of an activated anatase TiO_2_ aggregate powder (a) after 150 min of exposure to atomic hydrogen, (b) after 150 min under high-vacuum conditions in the absence of atomic hydrogen and (c) after the addition of 20 mbar O_2_. (d–f) Diffuse reflectance Vis spectra of an activated anatase TiO_2_ aggregate powder (d) after 150 min of exposure to atomic hydrogen, (e) after the addition of 0.1 mbar H_2_O for 3 min and reestablishment of high-vacuum conditions for 150 min and (f) after the addition of 20 mbar O_2_. Spectra of the activated aggregates (i.e., before chemical reduction) were used as the reference.

**Figure 2:**
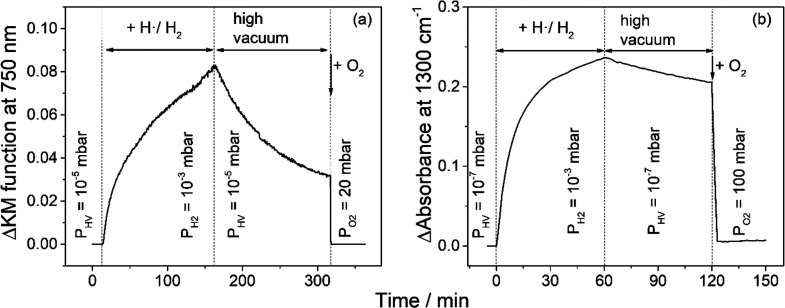
Evolution of the Kubelka–Munk function recorded at 750 nm for an activated anatase TiO_2_ aggregate powder (a) and of the absorbance recorded at 1300 cm^–**1**^ for an activated anatase TiO_2_ aggregate layer (b) upon exposure to atomic hydrogen, subsequent reestablishment of high-vacuum conditions, and final addition of molecular oxygen.

Sample exposure to atomic hydrogen induces significant changes in the Vis spectrum of an anatase TiO_2_ aggregate powder (Figure 1a). In particular, a broad absorption in the visible range (380 nm < λ < 800 nm) with a maximum at λ ∼ 780 nm evolves and saturates after ∼150 min (Figure 2a). This signal indicates the formation of Ti^3+^ centers upon sample reduction by atomic hydrogen (eq 2). Discontinuation of atomic hydrogen exposure and sample storage under high-vacuum conditions (*p* < 10^–5^ mbar) for 150 min lead to a decrease of the Vis absorption by ∼60%, while the signal envelope remains virtually unchanged (Figures 1b and 2a).

Molecular oxygen acts as an efficient electron scavenger when added to the gas phase leading to an immediate quenching of the Vis absorption (Figures 1c and 2a).

The significant decrease of the Ti^3+^ signal intensity, which is observed, when the surrounding H_2_/H· atmosphere is replaced by high vacuum (with a rest gas pressure of *p* < 10^–5^ mbar) may possibly result from the desorption of molecular hydrogen. Such a process would correspond to the reversal of reactive hydrogen uptake involving the recombination of electron/proton centers at the particle surface according to:
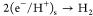
4

The corresponding second order rate law is
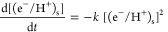
5

Integration yields the concentration of electron/proton centers at the semiconductor surface [(e^–^/H^+^)_s_] as a function of time t
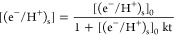
6

Here, [(e^–^/H^+^)_s_]_0_ is the (undetermined) initial concentration of electron/proton centers, and *k* is the corresponding second order rate constant.

The (first-order) desorption of hydrogen atoms, on the other hand, would give rise to an exponential decay of electron/proton centers according to
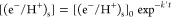
7with *k’* being the corresponding first-order rate constant. Equation 7 would also describe a possible (pseudo-first-order) proton-coupled electron transfer to acceptor molecules (e.g., oxygen) in the gas phase, provided that their concentration stays constant over time. In such a case, *k’* would correspond to the pseudo-first-order rate constant and its value would depend on the concentration of acceptor molecules.

**Figure 3:**
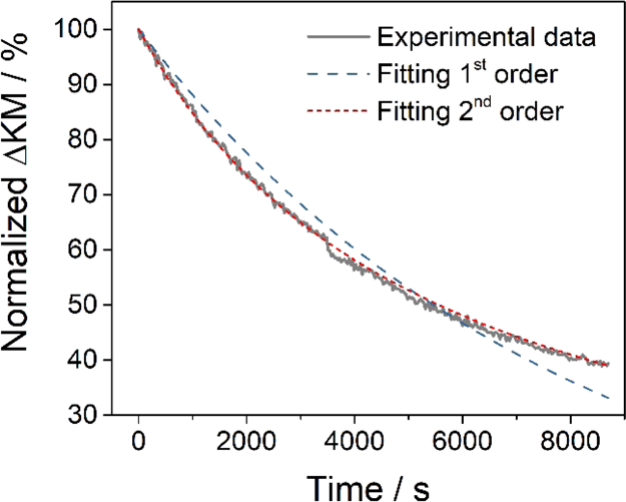
Fitting of the Vis-signal decay (corresponding to the normalized Kubelka–Munk function at 750 nm) observed for a chemically reduced anatase TiO_2_ aggregate powder upon reestablishment of high-vacuum conditions (compare Figure 2a). Fitting curves corresponding to a first-order decay (blue long-dashed line) and a second order (red short-dashed line) were used to fit the experimental data (gray solid line).

Equations 6 and 7 were fitted to the experimental data of the Vis-signal intensity decay as recorded upon the replacement of the H_2_/H· atmosphere under high-vacuum conditions (Figure 3). While the integrated rate law corresponding to the second order recombination of electron/proton pairs at the semiconductor surface (eq 6) closely resembles the observed intensity decay, experimental data significantly deviate from first-order kinetics (eq 7).

The good fit of the second order decay curve to the experimental data (which cover 60% of the total conversion, Figure 3) points to desorption of molecular hydrogen (eq 4) as the predominant process contributing to the depletion of the Vis-active electron center, which is observed when a H_2_/H· containing atmosphere is replaced by high-vacuum conditions. The recombination of H^+^/e^–^ pairs at the semiconductor surface and the subsequent desorption of molecular hydrogen correspond to the reversal of the chemical sample reduction by H atoms. Obviously, there exists a chemical equilibrium between hydrogen atoms in the gas phase and—following their dissociation—Ti^3+^/proton centers in the oxide. The equilibrium concentration of excess electrons in the semiconductor seems to depend critically on the concentration of atomic hydrogen in the gas phase.

#### 3.1.2 Reactivity of Ti^3+^ Centers in the Presence of Interfacial Water

Addition of water vapor to the gas phase results in the formation of a thin water layer at the oxide surface, which persists even upon the subsequent reestablishment of high-vacuum conditions (see Section 3.1.4). Interfacial water, importantly, does not significantly influence the evolution of the Vis spectrum of an aggregate powder previously reduced by atomic hydrogen (Figure 1d). In particular, exposure of the reduced aggregate powder to water vapor (*p*[H_2_O] = 0.1 mbar) for 3 min and subsequent sample storage for 150 min under high-vacuum conditions entails a decrease of the signal intensity in the visible by about 40% (Figure 1e). While the decrease of the signal intensity is comparable in the absence (Figure 1b) and in the presence (Figure 1e) of an interfacial water layer, a shift of the absorption maximum from λ ∼ 780 nm to λ ∼ 700 nm is observed in the latter case pointing to a location of Ti^3+^ species at the oxide surface. While interaction of surface Ti^3+^ with water dipoles may induce a minor modification of electron transition energies, our results clearly highlight the absence of an interfacial electron transfer from the semiconductor to water or its adsorption products at the oxide surface. However, the addition of oxygen (*p*[O_2_] = 20 mbar) leads again to a complete quenching of the Ti^3+^ signal and the initial reflectance of the powder is restored (Figure 1f). A very similar behavior was observed for anatase TiO_2_ aggregate films deposited onto FTO-coated glass substrates (Figure S1).

#### 3.1.3 Chemical Reduction of TiO_2_ Aggregate Layers by Atomic Hydrogen: IR-Active Conduction Band Electrons

IR spectra of activated anatase TiO_2_ aggregate layers feature sample-specific bands in two separate spectral regions. At least five overlapping, but clearly distinguishable bands contribute to the spectrum between 3800 and 3500 cm^–1^ (Figure S2a) and are assigned to the stretching vibration of isolated surface hydroxyl groups (Ti–OH groups).^34^ The irregular surface of TiO_2_ nanoparticles gives rise to different local geometries of the Ti–OH groups and, thus, to different vibrational frequencies.

The spectral range between 1800 and 1300 cm^–1^ (Figure S2b) features weak bands at 1686, 1621, 1572, 1525, 1462, 1380, and 1360 cm^–1^. This spectral region contains contributions from antisymmetric ν_as_(COO) and symmetric ν_s_(COO) stretching vibrations as well as δ(CH_3_) and the δ(HOH) bending vibrations.^35−37^ The observed bands are thus assigned to carbonate and carboxylate species.

The IR spectra clearly evidence the presence of surface remnants (chemisorbed water, organic adsorbates) after the activation process. Clearly, higher processing temperatures (i.e., *T* > 450 °C) under high-vacuum conditions and/or in an oxygen atmosphere would be necessary to desorb and decompose these surface species and generate adsorbate-free (i.e., clean) oxide surfaces. Such elevated processing temperatures, however, may induce significant morphological changes (e.g., particles growth and particle neck formation) and were, therefore, omitted.

**Figure 4:**
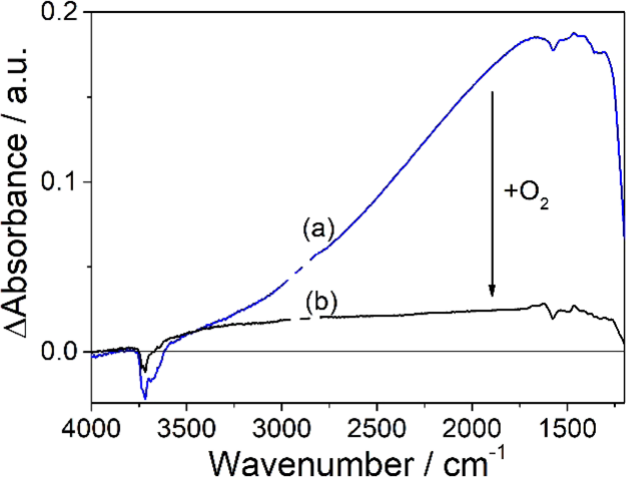
IR spectra of an activated anatase TiO_2_ aggregate layer (a) after 60 min of exposure to atomic hydrogen and (b) after storage for 30 min in an oxygen atmosphere (*p*(O_2_) = 100 mbar). Spectra are referred to the single channel spectrum of an activated aggregate layer.

Upon atomic hydrogen exposure, a Drude-type absorption characteristic of free conduction band electrons appears in the difference spectrum of the aggregate layer (Figure 4a). The structureless absorption increases monotonically toward lower wavenumbers between 3600 and 1550 cm^–1^ with a sharp cutoff near 1250 cm^–1^.

The temporal evolution of the signal intensity upon (i) atomic hydrogen exposure, (ii) subsequent reestablishment of high-vacuum conditions, and (iii) final addition of oxygen is tracked at 1300 cm^–1^ (Figure 2b). The signal intensity saturates after 60 min of sample exposure to atomic hydrogen. Reestablishment of high-vacuum conditions leads to a gradual decrease of the signal intensity. In particular, an intensity decrease of ∼15% is observed after sample storage for 60 min under high-vacuum conditions (Figure 2b). As in the case of Ti^3+^ centers, we attribute the slow depletion of the IR signal to the recombination of electron/proton centers at the oxide surface and hydrogen desorption.

Oxygen addition leads to an immediate and significant quenching of the IR signal (Figure 2b). However, ∼10% of the initial intensity remain even after 30 min of sample storage in an oxygen atmosphere (Figure 4b) pointing to an incomplete interfacial electron transfer to oxygen.

Obviously, a fraction of conduction band electrons remains in the semiconductor even in the presence of the electron acceptor. This unreactive fraction of electrons cannot be detected by Vis spectroscopy where oxygen addition leads to a complete quenching of the Vis absorption corresponding to Ti^3+^ centers (Figures 1 and 2a). The unreactive fraction of conduction band electrons is assigned to electron/proton pairs in the semiconductor bulk. Because of slow proton diffusion in the oxide bulk, these centers give rise to a transient n-type doping of the semiconductor. Interfacial electron transfer from electron/proton centers located at the surface, in contrast, is not limited by the diffusion of protons or hydrogen atoms in the oxide bulk and is, therefore, fast.

**Figure 5:**
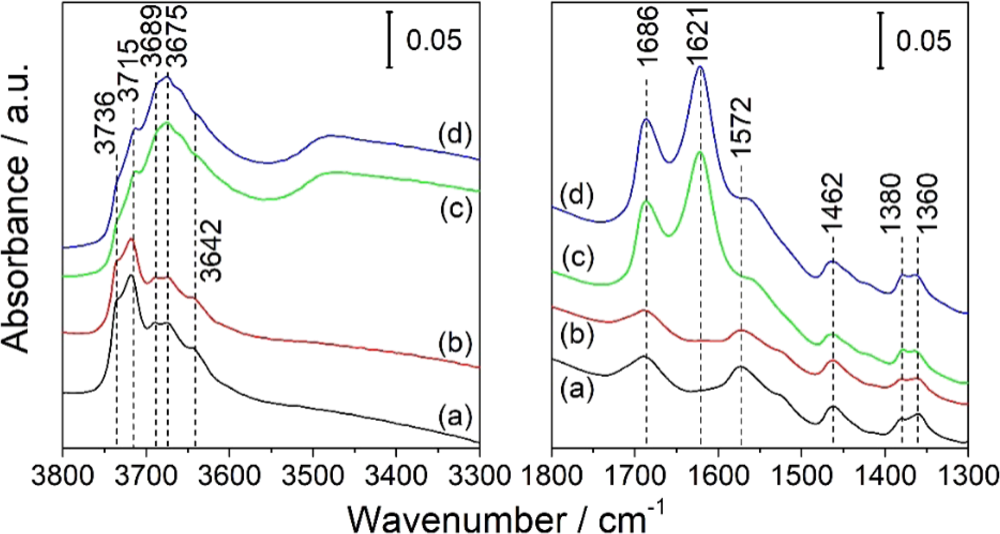
IR spectra of an activated TiO_2_ aggregate layer (a) before and (b) after chemical reduction with atomic hydrogen (60 min), (c) after subsequent water addition (0.1 mbar H_2_O, 3 min), and reestablishment of high-vacuum conditions (*p* = 10^–7^ mbar, 57 min) and (d) after final storage for 30 min in an oxygen atmosphere (*p*(O_2_) = 100 mbar). Spectra are referred to the single channel spectrum, which was recorded under high-vacuum conditions and with the sample/mesh removed from the IR path (empty beam).

IR bands assigned to the stretching vibration of OH groups on the surface of activated TiO_2_ aggregate layers experience a small (∼20%) decrease in intensity upon chemical reduction with atomic hydrogen (Figures 4 and 5a,b). However, the signal envelope in the corresponding wavenumber range remains unchanged. More importantly, upon the addition of oxygen and the associated interfacial electron transfer, the initial intensity of IR bands is restored (Figure 4). This minor (and reversible) change of the intensity of OH bands is attributed to a change of the respective extinction coefficients in the presence of excess electrons. Similar observations were made by Panayotov et al.^38^ upon the photooxidation of methanol on TiO_2_ nanoparticles. In that study, the spectral features associated with adsorbed methoxy groups were observed to decrease upon UV exposure, resulting in negative changes in IR absorptivity. At the same time, an increase of the background signal attributed to conduction band electrons was observed. The decrease of the intensity of methoxy-specific IR bands was nearly completely recovered in the absence of UV photons. The effect was assigned to electric field changes within the particles, rather than significant chemical reactions or desorption processes and was attributed to a Stark effect induced by trapped carriers.^39^

Chemical reduction of the aggregate layer by atomic hydrogen does not induce any displacement of IR bands (Figure 5a,b). This is in line with observations made by Yates and Panayotov,^34^ who did not observe any change in the IR-band positions of isolated OH groups on the surface of TiO_2_ nanoparticles upon thermal sample reduction (i.e., lattice oxygen removal) and the associated accumulation of excess electrons.

Finally, it has to be mentioned that the exposure of TiO_2_ aggregate layers to atomic hydrogen does not lead to an increase of the concentration of OH groups at the oxide surface. Furthermore, there is no indication of the formation of molecular water upon the chemical reduction of the oxide (Figure 5a,b).

#### 3.1.4 Reactivity of Conduction Band Electrons in the Presence of Interfacial Water

**Figure 6:**
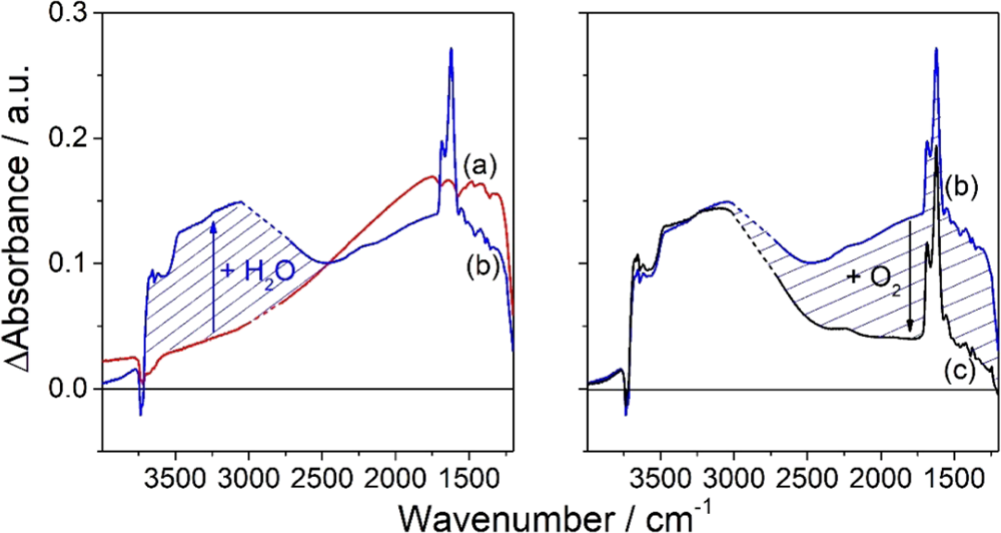
IR spectra of an activated anatase TiO_2_ aggregate layer (a) after 60 min of exposure to atomic hydrogen, (b) after subsequent addition of 0.1 mbar H_2_O for 3 min and reestablishment of high-vacuum conditions for 57 min and (c) after storage for 30 min in an oxygen atmosphere (*p*(O_2_) = 100 mbar). Spectra are referred to the single channel spectrum of an activated aggregate layer.

The presence of water induces significant changes in the IR spectrum of a chemically reduced anatase TiO_2_ aggregate layer (Figures 6a,b and 5b,c). In particular, a broad absorption extending from 3700 cm^–1^ to 2500 cm^–1^ as well as a narrow band at 1621 cm^–1^ appear in the spectrum after the addition of water vapor (*p* [H_2_O] = 0.1 mbar, 3 min) and the subsequent reestablishment of high-vacuum conditions for 57 min (Figures 6b and 5c).

The broad IR signal between 3700 and 2500 cm^–1^ as well as the narrow band at 1621 cm^–1^ are attributed to the stretching vibration of hydrogen-bonded OH groups and to the bending mode of molecularly adsorbed water, respectively. A closer inspection of the wavenumber range between 3750 and 3600 cm^–1^ reveals a broadening of bands corresponding to the stretching vibration of isolated OH groups as well as a change in the spectral envelope upon water adsorption (Figure 5b,c). Hydrogen bonding may involve surface OH groups as well as physisorbed water.

Apart from the IR band at 1686 cm^–1^, which experiences a narrowing of the band width, bands detected both for activated as well as for chemically reduced TiO_2_ aggregate layers between 1800 and 1300 cm^–1^ (and which are assigned to carbonate and carboxylate species) do not experience significant changes upon water addition (Figure 5b,c). The appearance of the intense band at ∼1621 cm^–1^ is associated with the physisorption of molecular water.^34^ While for liquid bulk water, the H–O–H dangling vibration gives rise to a band at 1640 cm^–1^,^40^ interfacial water, which is strongly interacting with the TiO_2_ surface, gives rise to a band at 1621 cm^–1^.^34,41,42^ It has to be emphasized that even 60 min after the establishment of high-vacuum conditions (via evacuation of the water vapor at room temperature), molecularly adsorbed water molecules remain adsorbed at the oxide surface, possibly forming a thin water film.

The monotonic absorption background corresponding to conduction band electrons experiences only a minor decrease in intensity upon water addition (Figure 6b). Obviously, conduction band electrons (as well as Ti^3+^ centers, compare Figure 1d,e) are unreactive toward water: 80% of the original signal intensity (corresponding to the broad background absorption) persist even 60 min after the addition of water vapor to the chemically reduced sample (Figure 6b). The decrease of the signal intensity by ∼20% is comparable to the intensity loss registered for a chemically reduced sample under high-vacuum conditions (Figure 2b) and is attributed to the desorption of molecular hydrogen.

The stability of conduction band electrons toward water differs from previous observations by Yates and co-workers.^34^ Concretely, these authors evidenced the withdrawal of conduction band electrons from thermally reduced TiO_2_ nanoparticles (Degussa P25, 70% anatase and 30% rutile) upon water addition. Ti–OH surface species resulting from water dissociation at oxygen vacancy sites were assumed to facilitate excess electron depletion. As discussed above, H atom dissociation at the TiO_2_ surface (as exploited in the present study) allows for electron accumulation upon preservation of the metal-to-oxygen ratio in contrast to thermal reduction of the oxide, which is associated with lattice oxygen removal. Obviously, water adsorption and consecutive charge transfer reactions are critically influenced by the presence of surface defects giving rise to significant differences in the reactivity of excess electrons generated in TiO_2_ nanoparticle systems by thermal reduction (lattice oxygen removal) or chemical reduction (H atom dissociation), respectively. The elucidation of the impact of different defect types on water chemistry at reduced TiO_2_ surfaces is highly relevant for applications such as solar water splitting.^43^ The experimental strategy reported in this paper may contribute to an advancement of analytical methodologies facilitating such an in depth understanding of interfacial reactions on highly dispersed semiconductor oxide systems.^44^

A major fraction of conduction band electrons persists in the presence of an interfacial water layer on the surface of chemically reduced TiO_2_ aggregates (Figure 6b). A similar behavior was found for Ti^3+^ centers (Figure 1d,e). However, while Ti^3+^ centers are quantitatively quenched in the presence of oxygen both in the absence and in the presence of interfacial water (Figure 1), a slightly different reactivity is observed for IR-active conduction band electrons. The addition of oxygen (*p*[O_2_] = 100 mbar, 30 min) to a chemically reduced aggregate layer in the presence of interfacial water leads to an intensity decrease of the broad background absorption by ∼85%. At the same time, the width of the bands at 1686 and 1621 cm^–1^ remains unchanged, while their intensity increases (Figures 6c and 5c,d). This resembles the (reversible) decrease of the intensity of OH bands in the presence of conduction band electrons (Figure 4). The broad absorption between 3600 and 2700 cm^–1^ corresponding to hydrogen-bonded OH groups as well as bands between 3750 and 3650 cm^–1^ corresponding to isolated surface OH groups remain virtually unchanged upon the addition of oxygen (Figures 6c and 5c,d). The absence of significant changes in the envelope of OH bands differs from findings on reduced TiO_2_ anatase (101) single crystal surfaces, where the formation of terminal OH groups was observed upon the reaction between water and oxygen.^45^ This discrepancy may result from the very high water activity in the experiments reported here. Under these conditions, the oxide surface will most probably be fully saturated with OH groups already prior to oxygen addition. Furthermore, very different defect sites are involved in nanoparticle-based systems (chemically reduced by H atoms) and surfaces of (mineral) single crystal surfaces. Notably, it was found that Nb impurities play an important role in the formation of the terminal OH groups.^45^

The depletion of the monotonic background absorption (Figure 6c) results from an interfacial electron transfer to oxygen, which is obviously not impeded by the presence of adsorbed water. However, the IR absorption corresponding to conduction band electrons is not completely quenched upon the addition of oxygen. A fraction of ∼15% persists even after sample storage for 30 min in an oxygen atmosphere. This resembles the behavior of chemically reduced TiO_2_ aggregate layers upon oxygen addition in the absence of physisorbed water (Figure 4) and points to a persistent n-type doping (i.e., hydrogen doping) of the oxide.

### 3.2 Electrochemical Charge Accumulation and Electron Transfer at the Solid/Electrolyte Interface

**Figure 7:**
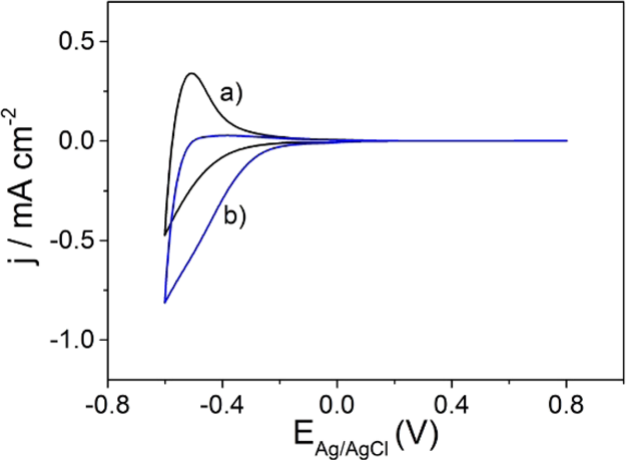
Cyclic voltammograms for an anatase TiO_2_ aggregate electrode measured in (a) N_2_- and (b) O_2_-purged 0.1 M HClO_4_ aqueous solution. Scan rate: *v* = 20 mV·s^–1^.

Cyclic voltammetry has proven particularly useful for the characterization of electron centers in anatase TiO_2_ nanoparticle films and for the evaluation of their reactivity at the solid/electrolyte interface.^4,27,46^ To extend the investigation of the reactivity of electron/proton centers from the solid/gas interface to the solid/electrolyte interface we recorded cyclic voltammograms (CVs) of anatase TiO_2_ aggregate electrodes in 0.1 M HClO_4_ aqueous electrolyte (Figure 7).

If the electrolyte is purged of residual oxygen by bubbling N_2_ through the solution, reversible currents are observed at potentials *E*_Ag/AgCl_ < −0.2 V (Figure 7a).^4,27,46^ The symmetrical shape of the CVs is indicative of capacitive processes associated with reversible electron accumulation (in the negative-going scan) and extraction (in the positive-going scan) at the semiconductor/electrolyte interface according to eq 1.

Despite difficulties associated with the definition and the exact experimental determination of the conduction band edge in nanosized systems,^3^ it is well-established that anatase TiO_2_ nanocrystal films feature an exponential surface state distribution just below the conduction band giving rise to capacitive currents in the CVs.^3,4,46^ The reversibility of these currents in the absence of oxygen (Figure 7a) evidences the absence of any significant interfacial electron transfer (i.e., the absence of Faradaic reactions). Accordingly, electrochemically accumulated electrons are inactive with regard to an interfacial electron transfer to (and thus the reduction of) water or its adsorption products. This inactivity can be rationalized by thermodynamic reasons, namely, the low reducing power of trapped electrons.

In the presence of appropriate electron acceptors in the electrolyte, however, electron transfer across the semiconductor/electrolyte interface may take place and the resulting Faradaic currents can be tracked by cyclic voltammetry. In this context, the electrochemical reduction of oxygen at the semiconductor/electrolyte interface has been used previously as a model reaction to investigate the reactivity of electrons trapped at band gap states in anatase TiO_2_ nanoparticle electrodes.^47^

Indeed, Faradaic currents are measured for anatase TiO_2_ aggregate electrodes at potentials *E*_Ag/AgCl_ < −0.2 V in the presence of dissolved oxygen (Figure 7b). Under these experimental conditions, charge transfer across the solid/electrolyte interface occurs in addition to capacitive processes. The observed asymmetrical shape of the CV therefore results from the superposition of a negative current density (both in the negative and positive scan direction) resulting from electron transfer to dissolved oxygen (Faradaic reaction) and of a negative (in the negative scan direction) or positive current density (in the positive scanning direction) resulting from capacitive charging/discharging of the semiconductor.

The appearance of Faradaic currents clearly demonstrates that the electron transfer from band gap traps in the semiconductor to dissolved oxygen is feasible not only at the semiconductor/gas interface (Section 3.1) but also at the semiconductor/electrolyte interface pointing to the favorable energetic location of band gap traps with respect to the O_2_/HO_2_·redox couple.^48^

Upon voltammetric cycling (Figure 7), electron accumulation and compensation take place mainly at the oxide surface. However, previous spectroelectrochemical studies have reported the appearance of a Drude-type IR-absorption (characteristic for conduction band electrons) and a broad Vis absorption (characteristic of Ti^3+^ centers) upon prolonged cathodic polarization of anatase TiO_2_ electrodes in acidic aqueous electrolytes.^17,23,24^ While the Vis signal showed a complete reversibility with respect to electrochemical charge extraction at positive potentials, the IR signal partially persisted even after prolonged polarization times.^15,16^ Based on this observations, Vis-active centers were assigned to localized Ti^3+^ species at the TiO_2_ particle surface and IR-active centers to shallow H^+^/e^–^ traps located at least partially in subsurface regions, giving rise to a persistent electrochemical doping of the electrode.^15,16^ These conclusions are perfectly in line with the conclusions drawn from our observations at the solid/vacuum interface (Section 3.1). Remarkably, chemical sample reduction by atomic hydrogen at the solid/vacuum interface resembles, at least to some extent, electrochemical sample reduction. A combination of model studies performed at different levels of complexity as proposed in the present study may therefore constitute a valuable tool for the identification of some physical and chemical properties influencing the materials’ functional properties under application-relevant conditions.

### 3.3 General Discussion

The chemistry of oxygen and water at the surface of highly dispersed metal oxide semiconductors is at the heart of technologically important processes such as the oxygen reduction reaction^49^ or water splitting.^43,50^ Therefore, great efforts are being made to gain molecular insight into underlying reaction steps on the one hand, and to identify the relationship between macroscopic measurables and interfacial properties on the other hand. This task is complicated by the fact that interfaces of working electro- and photocatalytic materials constitute highly complex systems.

Thermodynamic and kinetic details of the electron transfer from a particular semiconductor oxide particle to a particular acceptor species at the solid/gas or solid/electrolyte interface critically depend, for instance, on the type of exposed crystallographic faces,^51,52^ the presence of intrinsic^53^ or extrinsic defects,^45,54^ the presence of adsorbates^55,56^ or metal clusters,^56^ and the protonation state of functional surface groups.^57^ Furthermore, the experimental decoupling of the cascade beginning with electron generation and ending with the interfacial electron transfer is often challenging or even impossible. Importantly, most reaction steps critically depend on interfacial properties. In this regard, we believe that the methodology reported in this study will allow for investigating the reactivity of hydrogen-related electron centers at very different interfaces featuring partially tunable levels of complexity.

## 4 Conclusions

Aggregates consisting of anatase TiO_2_ nanoparticles were produced by a sequence of (i) MOCVS of isolated nanocrystals (*d* = 10–20 nm), (ii) purification of the resulting powder by thermal annealing under high-vacuum conditions and in an oxygen atmosphere, (iii) preparation of aqueous colloids, (iv) drying, and (v) final sintering of the samples in air. The resulting aggregates were investigated in the form of loose powders (by Vis spectroscopy), as layers immobilized on a tungsten mesh (by IR spectroscopy) and as electrodes, that is, films deposited onto a transparent conducting substrate (by cyclic voltammetry).

Powders and immobilized layers consisting of anatase TiO_2_ nanoparticle aggregates are chemically reduced by atomic hydrogen upon the formation of Vis-active Ti^3+^ centers and IR-active conduction band electrons. Excess electrons are slowly depleted under high-vacuum conditions. This process is assigned to the recombination of atomic hydrogen at the oxide surface and hydrogen desorption. The presence of an interfacial water layer does not significantly change the rate of excess electron depletion. Obviously, electron transfer from the semiconductor to water and its adsorption products is not feasible. While an interfacial transfer of conduction band electrons to surface water may be hindered kinetically, we attribute the inactivity of trapped electrons to thermodynamic reasons, that is, the low reducing power of trapped electrons. In the presence of molecular oxygen, Vis-active Ti^3+^ centers are immediately and completely quenched because of interfacial electron transfer reactions both in the presence and in the absence of an interfacial water layer. However, the addition of oxygen leads only to a partial consumption of IR-active conduction band electrons, and 10–15% of the initial IR signal intensity persists after 30 min in oxygen atmosphere pointing to a n-type doping of the sample upon exposure to atomic hydrogen.

The investigation of anatase TiO_2_ aggregate films as the electroactive electrode material reveals that electron transfer from the semiconductor to molecular oxygen is feasible not only at the solid/gas interface (as tracked by Vis- and IR-spectroscopy), but also at the solid/electrolyte interface (as tracked by cyclic voltammetry) because of the favorable energetic location of band gap traps with respect to the O_2_/HO_2_· redox couple. In the absence of dissolved oxygen, the semiconductor can be charged and discharged reversibly and no Faradaic currents are observed. This highlights the inactivity of electrochemically accumulated electron centers with regard to water reduction.

## Supporting Information Available

Additional Vis spectra of anatase TiO_2_ aggregate electrodes/films; IR spectra of anatase TiO_2_ aggregate layers; schematic drawings of the high-vacuum reactors; further experimental details (PDF)
