# Supplementary material for: Reactivity of Hydrogen-Related Electron Centers in Powders, Layers, and Electrodes Consisting of Anatase TiO2 Nanocrystal Aggregates
Source: J Phys Chem C Nanomater Interfaces. 2021 Jun 22;125(25):13809–18. doi: 10.1021/acs.jpcc.1c01580 (PMC8256420; doi:10.1021/acs.jpcc.1c01580)
Supplement: Supplementary file 1 — jp1c01580_si_001.pdf [file jp1c01580_si_001.pdf]

SUPPORTING INFORMATION

Reactivity of Hydrogen-related Electron Centers in Powders, Layers and Electrodes Consisting of Anatase TiO_2 Nanocrystal Aggregates

Juan Miguel Jiménez[a], Daniel Perdolt[a] and Thomas Berger[a,*]

[a] Department of Chemistry and Physics of Materials, University of Salzburg,

Jakob-Haringer-Strasse 2a, A-5020 Salzburg, Austria

E-mail: thomas.berger@sbg.ac.at

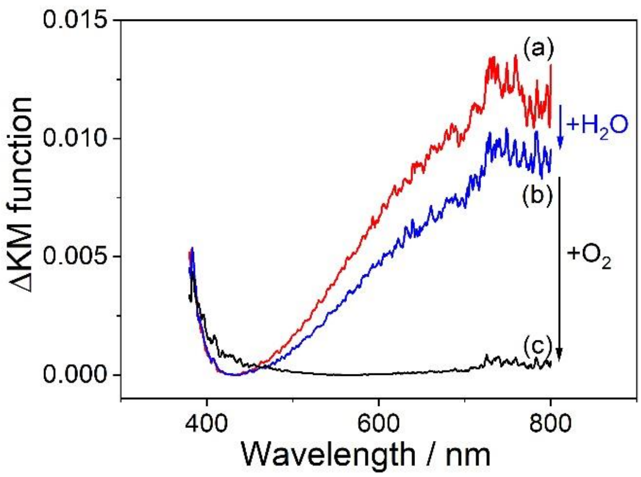

Figure S1: Diffuse reflectance Vis spectra of an activated anatase TiO_2 aggregate film (a) after 150 min of exposure to atomic hydrogen, (b) after addition of 20 mbar H_2O for 5 min and re-establishment of high vacuum conditions for 150 min and (c) after addition of 100 mbar O_2. The spectrum of the activated film was used as the reference.

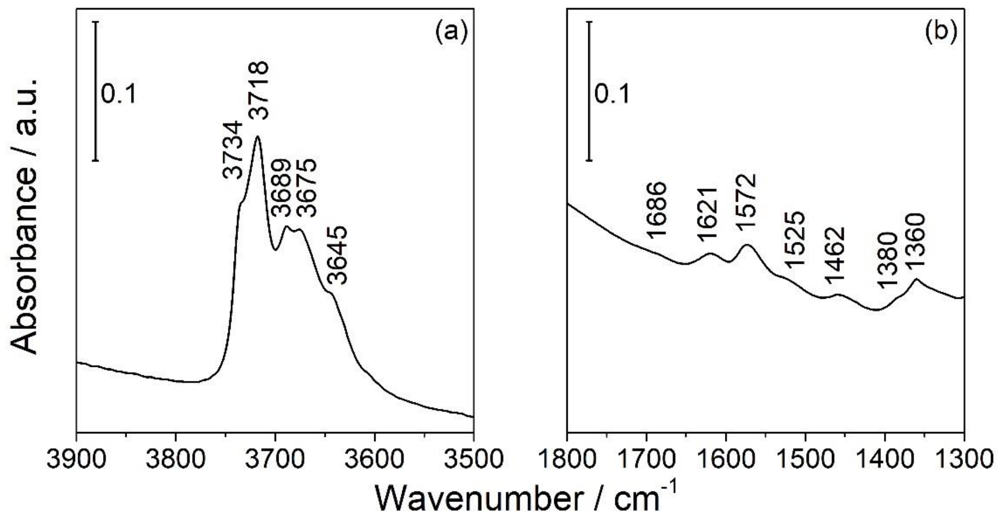

Figure S2: IR spectrum of an activated anatase TiO_2 aggregate layer. The spectral ranges feature (a) stretching vibrations of isolated OH-groups and (b) vibrations corresponding to carbonate and carboxylate species. The spectrum is referred to the single channel spectrum, which was recorded under high vacuum conditions and with the sample/mesh removed from the IR path (empty beam).

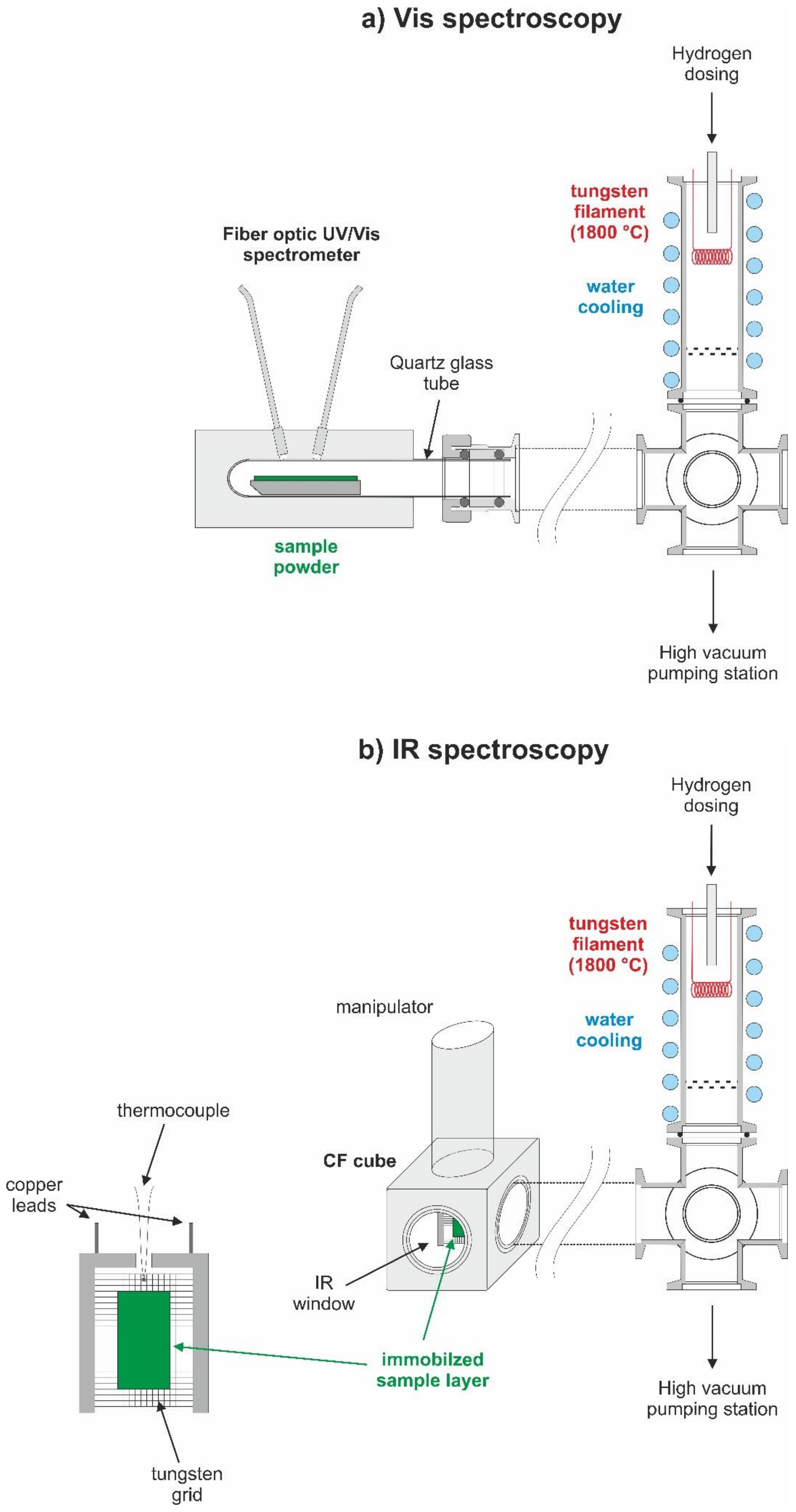

Figure S3: Schematic drawings of the high vacuum reactors used for chemical sample reduction by atomic hydrogen and consecutive reactions: (a) Vis-spectroscopic and (b) IR-spectroscopic experiments.

Further experimental details:

<u>High vacuum reactors for chemical sample reduction and spectroscopic experiments</u>

Due to the different geometry of Vis and IR spectrometer systems and of their corresponding sample cells it was not possible to use identical reactor geometries. However, great care was taken to design the high vacuum reactor in a way, which allows making geometrical conditions as comparable as possible. For this purpose, we used exclusively piping components with an inner diameter of 40 mm to connect the (identical) atomic hydrogen generator unit to the (geometrically significantly differing) sample cells (Figure S3). The separation distance between the tungsten filament and the sample cell accounts for ~45 cm in the case of UV/Vis-spectroscopic experiments (Figure S3a) and ~35 cm in the case of IR-spectroscopic experiments (Figure S3b).

While Vis spectra were recorded in reflectance mode, IR spectra were obtained in transmission mode. Therefore, different sample forms had to be investigated i.e. a powder or immobilized thin film (featuring sufficient reflectivity) for Vis experiments (Figure S3a) and a supported layer (of sufficient transmittance) for IR experiments (Figure S3b). However, as discussed in the Experimental Section of the manuscript, identical sample processing conditions were applied to yield nanoparticle aggregates featuring comparable properties.
